# HJURP promotes hepatocellular carcinoma proliferation by destabilizing p21 via the MAPK/ERK1/2 and AKT/GSK3β signaling pathways

**DOI:** 10.1186/s13046-018-0866-4

**Published:** 2018-08-15

**Authors:** Tianchi Chen, Hechen Huang, Yuan Zhou, Lei Geng, Tian Shen, Shengyong Yin, Lin Zhou, Shusen Zheng

**Affiliations:** 1 Division of Hepatobiliary and Pancreatic Surgery, Department of Surgery, First Affiliated Hospital, School of Medicine, Zhejiang University, Hangzhou, China; 2 Key Laboratory of Combined Multi-organ Transplantation, Ministry of Public Health, Hangzhou, China; 3 Key Laboratory of Organ Transplantation, Zhejiang Province, Hangzhou, China; 4 Key Laboratory of the diagnosis and treatment of organ Transplantation, CAMS, Hangzhou, China; 5 Collaborative Innovation Center for Diagnosis Treatment of Infectious Diseases, Zhejiang University, Hangzhou, China

**Keywords:** HJURP, Proliferation, p21, Hepatocellular carcinoma

## Abstract

**Background:**

Holliday junction recognition protein (HJURP) has been implicated in many cancers including hepatocellular carcinoma (HCC). However, the underlying mechanism by which HJURP promotes HCC cell proliferation remains unclear.

**Methods:**

RT-qPCR and immunohistochemistry were used to detect HJURP expression in HCC and adjacent tumor tissues and HCC cell lines. The localization of p21 were determined by immunofluorescence and western blot. Co-immunoprecipitation and western blot were used to validate the p21 stability and signaling pathways affected by HJURP. The effects of HJURP on HCC cell proliferation were assessed both in vivo and in vitro. The ERK1/2 pathway inhibitor U0126 and AKT pathway agonist SC-79 were used to treat HCC cell lines for further mechanistic investigations.

**Results:**

HJURP expression was higher in HCC tissues than in para-tumor tissues. Moreover, ectopic HJURP expression facilitated the proliferation of HCC cells, whereas the depletion of HJURP resulted in decreased cell growth in vitro and in vivo. Furthermore, the effects of HJURP silencing were reversed by p21 knockdown. Likewise, p21 overexpression inhibited cell growth ability mediated by HJURP elevation. Mechanistically, HJURP destabilized p21 via the MAPK/ERK1/2 and AKT/GSK3β pathways, which regulated the nucleus-cytoplasm translocation and ubiquitin-mediated degradation of p21. Clinically, high HJURP expression was correlated with unfavorable prognoses in HCC individuals.

**Conclusions:**

Our data revealed that HJURP is an oncogene that drives cell cycle progression upstream of p21 in HCC. These findings may provide a potential therapeutic and prognostic target for HCC.

## Background

Hepatocellular carcinoma (HCC) accounts for 70–75% of liver cancers and ranks as the third-most devastating malignancy, with a high risk of cancer-related death in the world [1]. Currently, hepatic resection and liver transplantation are the most effective surgical strategies for HCC, but the prognosis of HCC patients remains unsatisfactory with a 12% 5-year overall survival rate. Although substantial progress has been made in diagnostic and treatment strategies in recent decades, the high mortality and recurrence rates are still the major obstacles preventing the improvement of the curative effects of treatments for HCC [2]. Many molecular targeting agents have been investigated, but only sorafenib has exhibited limited efficacy in individuals with advanced HCC [3]. Therefore, it is important to identify a novel therapeutic target that will improve the prognosis of HCC patients.

Holliday junction recognition protein (HJURP) is a histone H3 chaperone that mediates CENP-A deposition at human centromeres during the early G1 phase [4]. In mammals, HJURP has been identified as a key factor of DNA binding and phosphorylation that promotes chromosomal segregation and cell mitosis [5]. In cancer, HJURP has been reported to be highly expressed in some malignancies, including HCC, breast cancer, lung cancer, bladder cancer and glioma [6–11]. Hu et al. found that HJURP promotes cell proliferation in vitro and that its expression is correlated with unfavorable clinical outcomes in HCC [9]. Moreover, Huang et al. demonstrated that a single nucleotide polymorphism (SNP) of HJURP, i.e.,rs3771333, can predict a higher susceptibility to HCC among Chinese people [12]. However, the underlying mechanism by which HJURP promotes HCC progression remains to be clarified.

p21 is a cyclin-dependent kinase inhibitor that is involved in cell cycle progression and DNA damage response. p21 can be degraded in either a ubiquitin-dependent or a ubiquitin-independent manner [13]. Additionally, the stability of p21 is regulated by several distinct mechanisms, including mechanisms involving extracellular signal-regulated kinase 1/2 (ERK1/2), glycogen synthase kinase (GSK) 3β and c-Jun N-terminal kinase (JNK) [14–16]. p21 is frequently dysregulated in cancer cells [17]. Because of the vital role of p21 in cancer development, it is important to understand the mechanisms underlying the dysregulation of p21 in HCC.

In this study, HJURP was found to facilitate cell proliferation in HCC both in vivo and in vitro. Additionally, HJURP functioned to at least partially mediate p21 nucleus-cytoplasm translocation. Furthermore, HJURP destabilized p21 by mediating ubiquitination. Overall, we provide evidence that HJURP promotes HCC cell proliferation by destabilizing p21 via the MAPK/ERK1/2 and AKT/GSK3β signaling pathways.

## Methods

### Clinical specimens

All of the HCC and adjacent tissues were from the First Affiliated Hospital, Zhejiang University School of Medicine, Zhejiang, China. This research was approved by the Ethical Review Committee of this hospital. Written informed consent was received according to the guidelines of the Declaration of Helsinki.

### Cell culture

Four human HCC cell lines (HCC-LM3, SMMC-7721 and Huh7) and one normal hepatocyte cell line (LO2) were obtained from the Cell Bank of the Shanghai Institutes of Biological Sciences, Chinese Academy of Sciences. HCC-LM3 and Huh7 cells were cultured in DMEM high glucose medium, and the SMMC-7721 and LO2 were cultured in RMPI 1640 medium (BI, Israel) with 10% fetal bovine serum (Biological Industries, Israel) in a 37 °C,5%CO_2_ incubator (Thermo Scientific, USA).

### Immunohistochemistry

Paraffin-embedded tissues were cut into 5 μm sections. The immunohistochemistry analysis was performed as we previously reported [18]. The primary antibodies against HJURP and Ki-67(Abcam, UK) were used at concentrations of 1:200.

### Lentivirus constructs and transfection

For HJURP down-regulation and up-regulation, lentivirus was purchased from GeneChem (Shanghai, China). Two HCC cell lines (Huh7 and HCC-LM3) were used for the knockdown assays, and SMMC-7721 cells were used for the overexpression experiment according to the instruction book. Stably transfected cells were selected via the application of 4 μg/ml puromycin (Sigma-Aldrich, USA) for 2 weeks.

### RT-qPCR

RNA was extracted with Trizol Regent (Thermo Scientific, USA). For the mRNA analysis, the TaKaRa PrimeScript RT Reagent Kit (TaKaRa, Japan) and the Bio-Rad QX100 Droplet Digital PCR system were used to conduct real-time quantitative PCR analysis. All premiers were obtained from Tsingke Biological Technology (Beijng, China). The primers were as follows: *HJURP* forward 5’-AGTGCCTTTATGTATTGGAG-3′, and reverse 5′- AAGTGAGGGTCTGGATTTA-3′; *p21* forward 5’-GCAGACCAGCATGACAGATT-3′, and reverse 5’-TAAGGCAGAAGATGTAGAGCG-3′; and *GAPDH* forward 5′- GAACATCATCCCTGCCTCTACT-3′, and reverse 5′- ATTTGGCAGGTTTTTCTAGACG-3′. *GAPDH* was used as an internal control.

### Western-blot

The total proteins were extracted for 60 min on ice in RIPA buffer (Thermo Scientific, USA) containing protease and phosphatase inhibitors (Cell Signaling Technology, USA). Cell lysates were centrifuged at 1.2 × 10^4^ g, 4 °C for 15 min, and the concentrations of supernatants were detected with a BCA Protein assay kit (Thermo Scientific, USA). 30 μg protein was separated by 10% SDS-PAGE (Life Technology, USA) and then transferred to 0.45 μm PVDF membranes (Millipore, USA). The membranes were incubated with monoclonal antibodies at 4 °C for 24 h. In total, primary antibodies included those for HJURP, ERK1/2, p-ERK1/2, cyclinD1, cyclinE, p-JNK, GSK3β, p-GSK3β AKT, p-AKT (Abcam, UK), LRR1 (Proteintech, China) and p21 (Cell Signaling Technology, USA). The immunoblots were detected with a visual imaging system (Bio-Rad, USA). β-actin and GAPDH (Solarbio Life Science, China) were selected as the loading controls.

### Cell viability assay

The cell viability assays were performed with a Cell Counting Kit-8 Assay (DOJINDO Laboratories, Japan). The HCC cells were seeded into 96-well plates (1 × 10^3^cells per well for the HCC-LM3 and SMMC-7721 cells, and 2 × 10^3^cells per well for the Huh7 cells) in 100 μl medium incubated at 37 °C, 5%CO_2_ in humidified incubator. After the indicated number of days, the supernatants were removed, 90 μl medium and 10 μl CCK-8 were added to each well, and the plates were then incubated for 1 h. The absorbance at 450 nm was detected with a microplate reader (BioTek, USA).

### Cell cycle analysis

The HCC cells were collected and fixed using 75% pre-cooled ethanol at 4 °C overnight. After being washed three times with phosphate buffered saline (PBS) and resuspended with 300 μl DNA staining solution (Multiscience, China) at room temperature for 30 min. The cell cycle analysis was detected via a flow cytometry (FACS LSRII, BD Bioscience, USA).

### Colony formation assay

For colony formation assessment, 2 × 10^3^ stably infected cells were seeded into 6-well plates. After incubation for 15 days, the plates were washed with PBS for three times and 4% paraformaldehyde used to fix the cells for 25 min. Subsequently, the cells were stained with 0.5% crystal violet solution for further counting and statistical analysis.

### Immunofluorescence assay

For immunofluorescence assay, 5 × 10^4^ stably transfected tumor cells were seeded in a 2 mm confocal plate (Nunc, USA) for culture in the indicated incubator. Cultured cells were fixed with 4% paraformaldehyde and then permeabilized with 0.25% TritonX-100. After blocking with 1% bovine serum albumin (BSA), the primary antibodies against p21 (1:100) (Abcam, USA) were added into each plate for an overnight incubation at 4 °C. The secondary antibodies (1:200, Sigma-Aldrich, USA) were incubated with the cells at 37 °C for 30 min and DAPI was used to stain the nuclei at 37 °C for 10 min. The images were captured by a fluorescence microscope (Olympus BX53, Japan).

### Nuclear and cytoplasmic protein extraction

To isolate the nuclear and cytoplasmic proteins of the HCC cells, a Nuclear and Cytoplasmic Extraction Reagents Kit (NE-PER™, Thermo scientific, USA) was used for the HCC cell lysis. Lentivirus infected HCC-LM3 and SMMC-7721 cells and their control groups were prepared for protein extraction. The detailed operating procedures were performed according to the instructions of manufacturer.

### Mouse xenograft assay

To assess the influence of HJURP on tumorigenesis in vivo, 4-week-old male BALB/c nude mice were purchased from Vital River Laboratory Animal Technology Co., Ltd. (Beijing, China). The mice were divided randomly into two groups and subcutaneously injected at 6-week-age with 4 × 10^6^ HCC cells per mouse. The nude mice of each group were sacrificed after 4 weeks for weight and volume measurements. The tumor volumes were calculated every 4 days using the following formula: volume (v) = (length×width^2^)/2. All animal experiments were approved by the Ethics Committee for Laboratory Animals of the First Affiliated Hospital, Zhejiang University School of Medicine, Zhejiang, China.

### Co-immunoprecipitation and ubiquitination assays

The cell samples were lysed in a IP buffer containing 1 mM DL-dithiothreitol (DTT), 100 mmol/L NaCl,1 mM MgCl_2_(Life Technology, USA) and protease inhibitor cocktails (Cell Signaling Technology, USA). The homogenates were incubated on ice for 45 min. Subsequently, the samples were centrifuged at 2600 g for 15 min at 4 °C. The total cell lysates were used for immunoprecipitation with p21 primary antibodies on protein A/G mix beads (Thermo Scientific, USA) overnight. The immunoprecipitates were collected and washed three times and prepared for Western-blot analysis. To detect p21 ubiquitination, 10 μΜ of the protease inhibitor MG132 (MedChem Express, USA) was added to the medium 5 h before lysis.

### Statistical analysis

The experiments were conducted in triplicates, and the data are presented as mean ± SD. Comparisons between groups were performed by Student’s *t* test. The overall survival rate curves of HCC patients based on Kaplan-Meier method were plotted using the log-rank test. The correlations between HJURP and the factors were assessed with χ^2^ test. *P* values < 0.05 were considered statistically significant, *represents *P* < 0.05, ** represents *P* < 0.01 and ***represents *P* < 0.001.

## Results

### HJURP is highly expressed in HCC cells and tissues

**Fig. 1:**
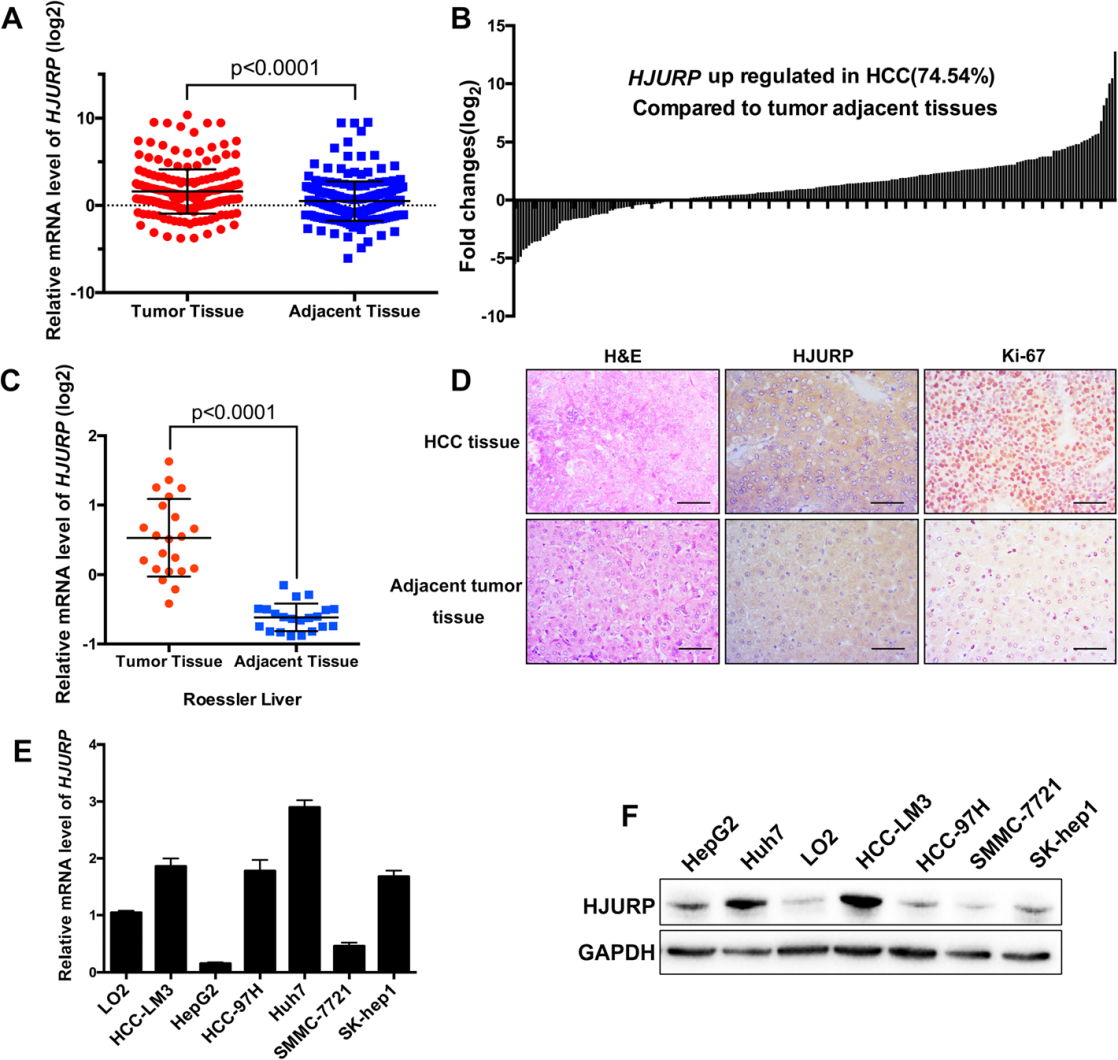
HJURP is overexpressed in HCC specimens and cell lines. **a** and **b** RT-qPCR analysis of HJURP in 219 clinical HCC samples and matched adjacent normal tumor samples. Compared to that in the matched para-tumor tissues, HJURP expression was upregulated in 74.54% HCC samples. **c** Expression of HJURP in Oncomine database. **d** Representative hematoxylin-eosin and IHC staining for HJURP in HCC tissues (scale bar: 50 μm; magnification: 200X). **e** mRNA level of *HJURP* in an immortalized hepatic cell line and six HCC cell lines. **f** Protein level of HJURP in six HCC cell lines and the LO2 cell line

We first detected the mRNA levels of *HJURP* in 219 pairs of HCC specimens and adjacent normal tissues by RT-qPCR. *HJURP* was more highly expressed in 74.54% of tumor tissues than in normal tissues (Fig. 1a, b). Additionally, data from the Oncomine database (Roessler liver) proved that *HJURP* expression was increased in HCC tissues (Fig. 1c). Similar results were observed regarding HJURP protein levels examined by immunohistochemistry (IHC) (Fig. 1d). Moreover, we extracted the total RNA and protein from the HCC cell lines to perform RT-qPCR and western blot. HJURP was apparently upregulated in HCC cell lines (Fig. 1e, f). These results indicated that HJURP might be correlated with HCC progression.

### HJURP promotes HCC tumor growth both in vivo and in vitro

To examine our hypothesis, we next examined the influence of HJURP on the biological behaviors of HCC cell lines. According to the expression levels, the HCC-LM3 and Huh7 cell lines were chosen for stable knockdown experiments using a lentivirus vector. Additionally, the SMMC-7721 cell line was selected for overexpression experiments.

**Fig. 2:**
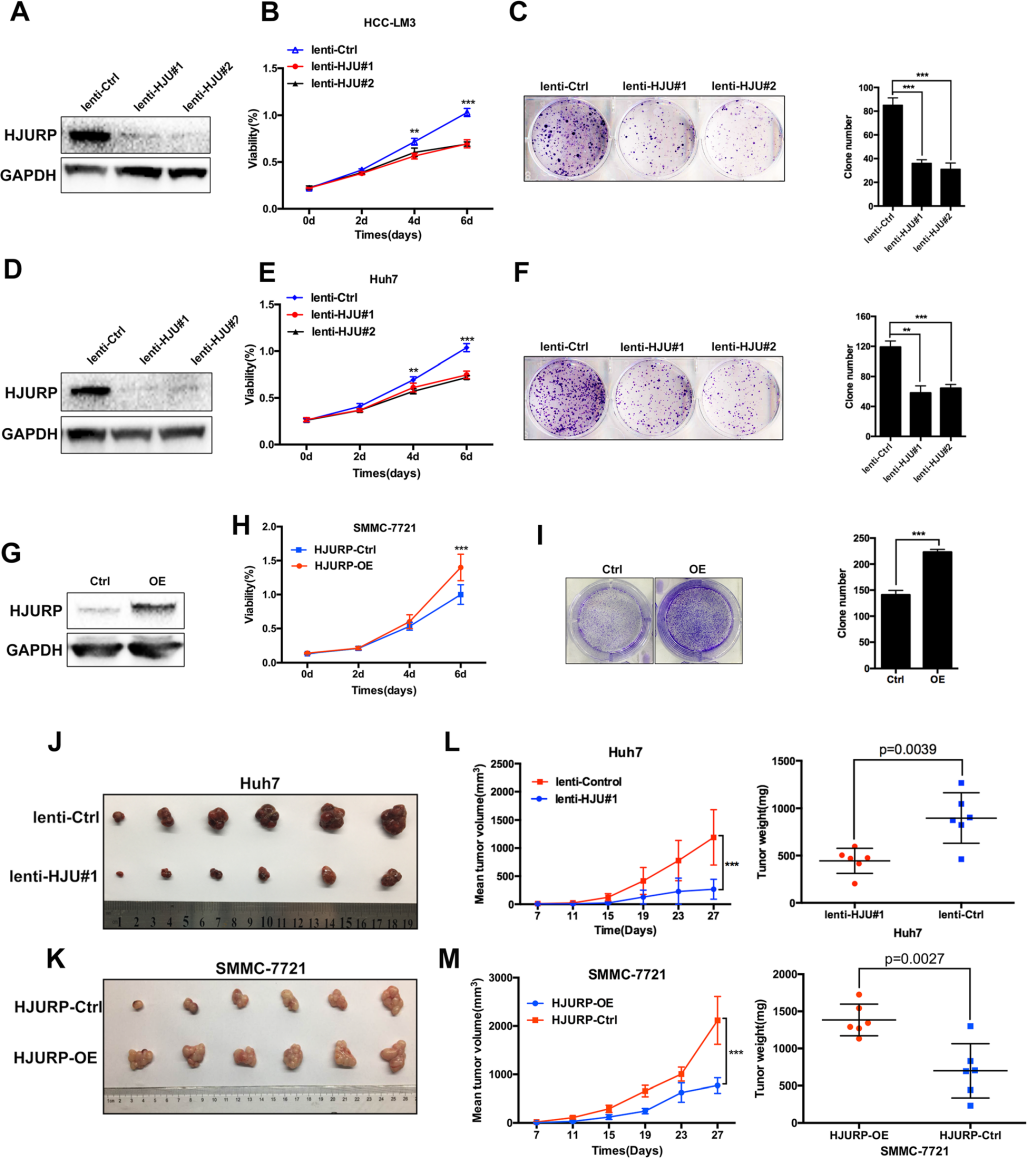
HJURP promotes HCC cell proliferation both in vivo and in vitro. **a** The efficiency of HJURP knockdown in HCC-LM3 cells. **b** Cell growth was detected by CCK-8 assays after 0, 2, 4 and 6 days (*n* = 8). **c** Colony formation of HCC-LM3 cells. **d** Efficiency of HJURP knockdown in Huh7 cells. **e** Cell growth was detected by CCK-8 assays in Huh7 cells after 0, 2, 4 and 6 days (*n* = 8). **f** Colony formation of Huh7 cells. **g** Efficiency of HJURP overexpression in SMMC-7721 cells. **h** Cell growth was detected by CCK-8 assays in SMMC-7721 cells. **i** Colony formation of SMMC-7721 cells. **j** and **k** HJURP facilitates the tumorigenicity of HCC cells in vivo. **l** and **m** Tumor volumes and weights were measured every 4 days. lenti-HJU#1: HJURP knockdown group#1; lenti-HJU#2: HJURP knockdown group#2; lenti-Ctrl: negative control group; HJURP-OE: HJURP-overexpressing group; and HJURP-Ctrl: control group

Subsequently, cell viability and colony formation assays were performed to examine the role of HJURP in tumor proliferation. Knockdown of HJURP dramatically suppressed cell growth and colony formation in HCC-LM3 (Fig. 2b, c) and Huh7 cells (Fig. 2e, f). Conversely, the ectopic expression of HJURP promoted cell growth and colony formation in SMMC-7721 cells (Fig. 2h, i).

To determine the function of HJURP in vivo, we subcutaneously injected BALB/c nude mice with stably transfected Huh7 cells. After 4 weeks, we found that the tumor volume and mean tumor weight were significantly lower in the HJURP knockdown group (lenti-HJURP#1) than in the negative control group (Fig. 2j, l). In contrast, the overexpression of HJURP in the SMMC-7721 cells markedly promoted tumor growth in vivo (Fig. 2k, m).

Collectively, these findings suggest that HJURP promotes HCC tumorigenesis both in vitro and in vivo.

### HJURP exerts a tumor-promoting effect by decreasing p21 expression and facilitating cell cycle progression in HCC

**Fig. 3:**
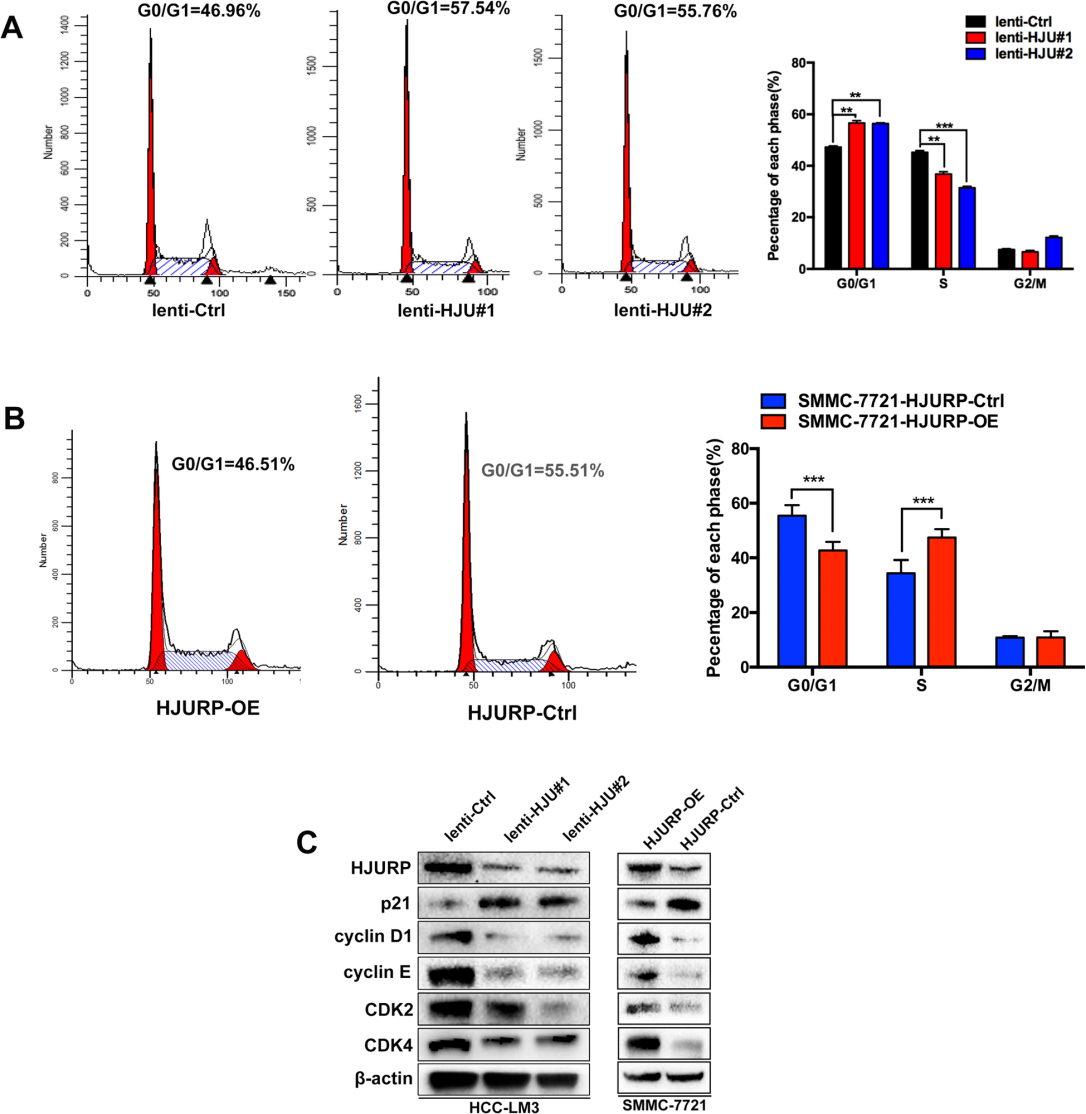
HJURP promotes HCC cell proliferation by regulating the cell cycle. **a** and **b** Cell cycle distribution was measured by flow cytometry in the HCC-LM3 and SMMC-7721 cells. **c** Cell cycle-related proteins including p21 were detected by western blot in the indicated cells

Consistent with the colony formation and cell viability assay results, we discovered that HJURP depletion generally induced G0/G1 arrest (Fig. 3a). In contrast, the promotion of G1/S phase transition was observed when HJURP was overexpressed (Fig. 3b). These results indicated that HJURP may mainly promote HCC tumor growth in the G0/G1 phase.

To verify our findings, we detected cell cycle checkpoint proteins in stably transfected cells using western blot. HJURP knockdown upregulated the tumor suppressor p21 and downregulated CyclinD1, CyclinE, CDK2 and CDK4 in HCC-LM3 cell (Fig. 3c, left). In contrast, HJURP overexpression downregulated p21 and upregulated the indicated checkpoint proteins in the G1 phase in SMMC-7721 cells (Fig. 3c, right).

**Fig. 4:**
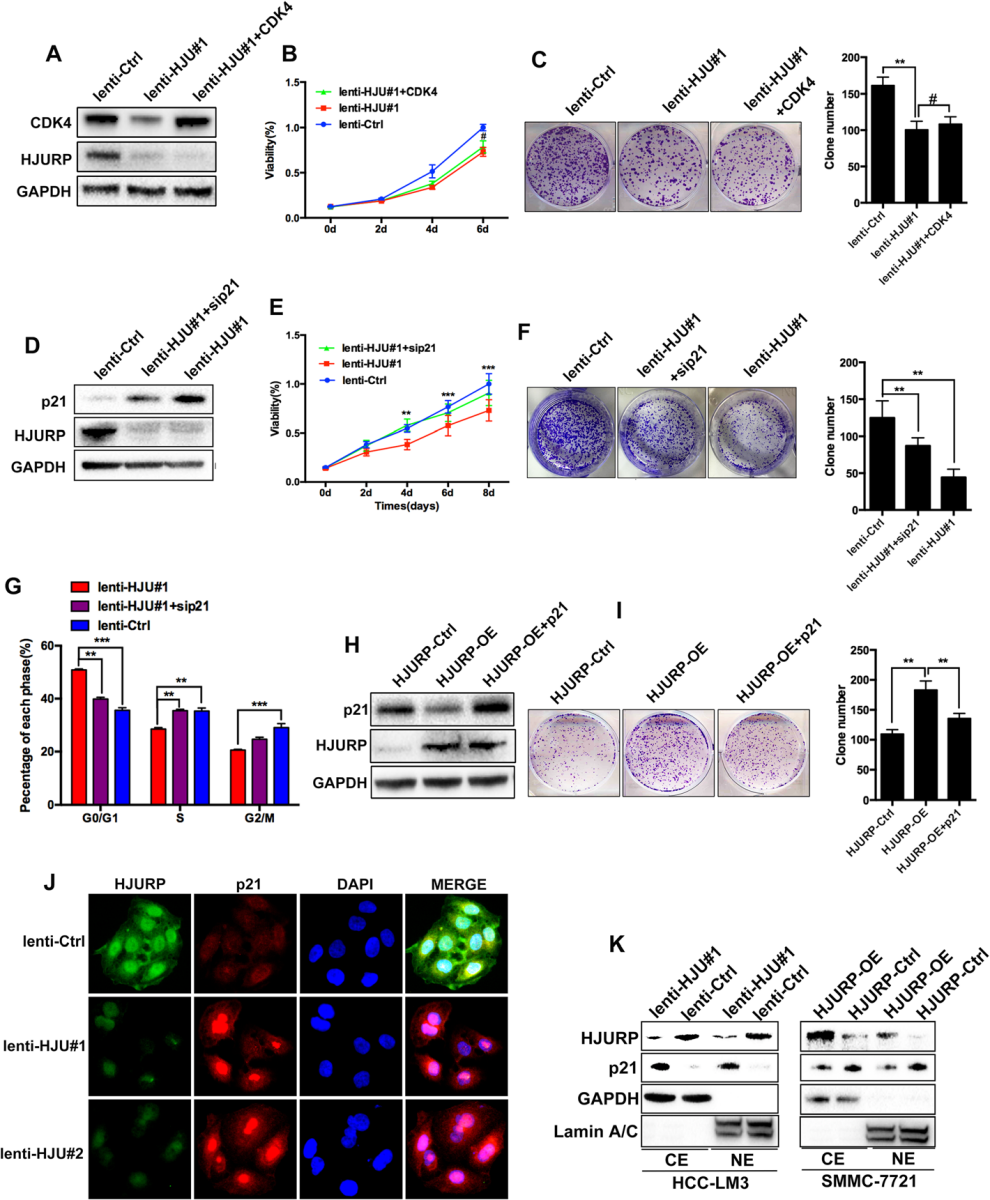
**a** CDK4 overexpression plasmids were transfected into lenti-HJU#1 cells. CDK4 protein expression was detected by western blot. **b** CCK-8 assay of lenti-HJU#1, lenti-Ctrl and lenti-HJU#1 + CDK4 group. **c** Colony formation assays of the indicated groups. Approximately 1500 cells/well were seeded and cultured in 6-well plates for 14 days. **d** Efficiency of p21 knockdown (sip21) in HCC-LM3 cells. **e** Silencing of p21 in the HJURP-knockdown HCC-LM3 cells rescued cell growth function in CCK-8 assay, **f** colony formation assay and **g** cell cycle assay. **h** p21 overexpression plasmids were transfected into HJURP-overexpressing (HJURP-OE) cells. Efficiency of p21 overexpression was determined by western blot. **i** p21 overexpression inhibited the colony formation ability of the HJURP-OE SMMC-7721 cells. **j** Expression of p21 was detected by immunofluorescence in the HCC-LM3 cells. **k** Nuclear p21 expression was detected in HCC cell lines. CE: cytoplasm. NE: nucleus

Because HJURP influenced the expression of two checkpoint proteins (p21 and CDK4) (Fig. 3c), we speculated that HJURP might promote cell cycle progression by mediating the p21 or CDK4 expression. To test our hypothesis, we used CDK4 overexpression plasmids in the lenti-HJU#1 group. Then, CCK-8 and colony formation assays were performed to determine the effect of CDK4 overexpression on HJURP knockdown cells. Surprisingly, CDK4 overexpression could not significantly reverse the growth of cells in the lenti-HJU#1 cells (Fig. 4a, b, c). Given that HJURP modulates tumor growth through p21, we depleted p21 expression in HJURP-silenced HCC cells and examined the colony formation and proliferation. The data revealed that p21 silencing reversed the tumor promoting activity of HJURP indicated by CCK-8 assays (Fig. 4e), colony formation assays (Fig. 4f) and cell cycle analysis (Fig. 4g). In accordance with this result, p21 overexpression inhibited colony formation in HJURP-overexpressing cells (Fig. 4h, i). In addition, based the immunofluorescence assay, we found that HJURP knockdown increased p21 expression in HCC-LM3 cells (Fig. 4j).

### HJURP promotes p21 nucleus-cytoplasm translocation in HCC cells

p21 has been recognized to play an important role in inhibiting the cell cycle, depending on its nuclear accumulation [19]. Moreover, the nucleus-cytoplasm translocation of p21 can initiate its degradation pathway [14]. In the immunofluorescence assay, stronger nuclear signals of p21 were detected in the HCC-LM3 cells when HJURP was knocked down (Fig. 4j). Furthermore, cytoplasmic and nuclear proteins were extracted and then examined by western blot. Notably, the nuclear and cytoplasmic expression of p21 was increased in the HJURP knockdown HCC-LM3 cells (Fig. 4k, left) and reduced in the HJURP-overexpressing SMMC-7721 cells (Fig. 4k, right).

These results demonstrated that HJURP promotes the nucleus-cytoplasm translocation of p21 in HCC cells.

### HJURP decreases the stability of p21 through an ubiquitin-dependent manner

**Fig. 5:**
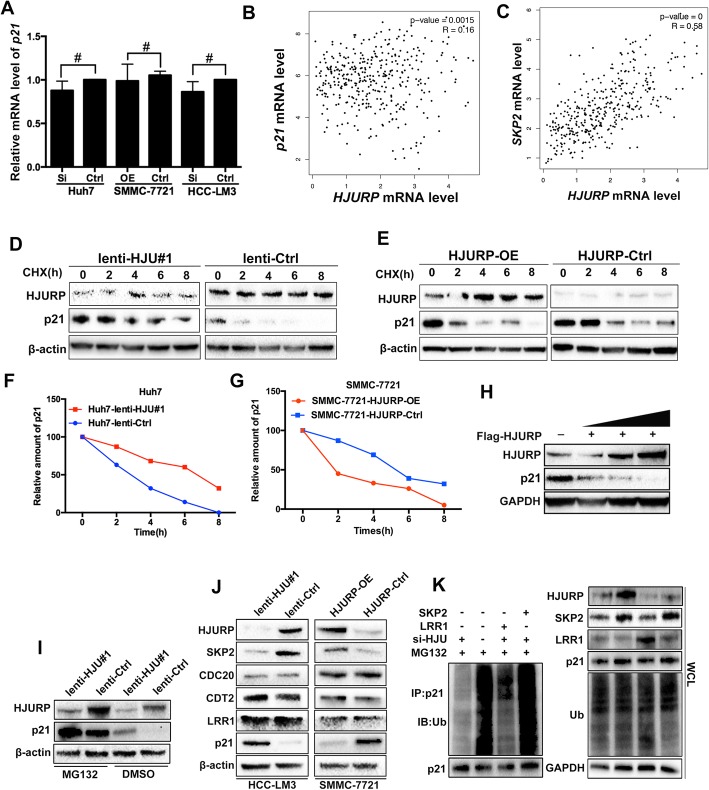
HJURP promotes HCC cell growth by inducing p21 ubiquitination. **a** RT-qPCR analysis of *p21* in HJURP knockdown and overexpressed HCC cell lines. *GAPDH* was selected as the internal control. **b** Scatter plots shows a negative correlation between *HJURP* and *p21* at the mRNA level. **c** *HJURP* was positively correlated with *SKP2* at the mRNA level. **d** and **e** HJURP decreased p21 stability. p21 was detected by western blot after cells incubated with CHX for the indicated times. **f** and **g** Relative amount of p21 protein expression in Huh7 and SMMC-7721 cells. The amounts of p21 were analyzed by ImageJ. **h** Following the increase in HJURP expression, p21 exhibited a decreasing trend in the SMMC-7721 cells. **i** Western blot was performed in HCC-LM3 cells transfected with HJURP knockdown lentivirus and treated with DMSO or MG132. **j** Four E3 ubiquitin ligases (SKP2, CDC20, LRR1 and CDT2) were detected by western blot. **k** Depletion of HJURP attenuated p21 ubiquitination. SKP2 and LRR1 overexpression plasmids were transfected into HJURP knockdown cells. Cells were treated with MG132 (10 μM) for 5 h before being harvested. Immunoprecipitation was used for the detection of p21 degradation

Because HJURP depletion increased p21 protein levels, we next asked whether HJURP affects the expression of p21 at the transcriptional or post-transcription level. To answer this question, we used RT-qPCR to detect the mRNA level of p21. Surprisingly, no significant differences were observed in the mRNA levels of the transfected Huh7, HCC-LM3 and SMMC-7721 cells relative to those in the respective controls (Fig. 5a). Meanwhile, gene expression data from The Cancer Genome Atlas (TCGA) indicated that the lack of correlation between the mRNA levels of *HJURP* and *p21*(Fig. 5b). However, *HJURP* expression was correlated with *SKP2*, which is an E3 ubiquitin ligase of p21 (Fig. 5c). Next, HCC cells were treated with a translation inhibitor, cycloheximide (CHX), at several specific time points. As expected, pretreatment with CHX resulted in a prolonged p21 half-life and a high relative amount in the HJURP knockdown Huh7 cells (Fig. 5d, f). In contrast, the ectopic expression of HJURP in SMMC-7721 cells led to a greater degradation of p21 than in control cells (Fig. 5e, g). Furthermore, following the increase in HJURP expression, p21 expression exhibited a decreasing trend in SMMC-7721 cells (Fig. 5h). Additionally, treatment with MG132 (a proteasome inhibitor) significantly reversed the HJURP-induced downregulation of p21 in HCC-LM3 cells (Fig. 5i).

To date, p21 has have been reported to be degraded by four E3 ubiquitin ligases, namely, SKP2, CDC20, CDT2 and LRR1 [20–23]. To determine the protein levels of these four E3 ubiquitin ligases, we extracted total protein from lentivirus-infected HCC-LM3 and SMMC-7721 cells for western blot analysis. As expected, SKP2 was downregulated in HJURP knockdown HCC-LM3 cells, whereas SKP2 expression was upregulated in the HJURP-overexpressing SMMC-7721 cells (Fig. 5j). Because *HJURP* was correlated with *SKP2*, we further investigated the ubiquitination of p21. In HCC-LM3 cells, HJURP knockdown decreased the ubiquitination of p21. However, SKP2 but not LRR1 overexpression increased p21 ubiquitination in HJURP knockdown cells (Fig. 5k).

Taken together, these data indicate that HJURP decreases the stability of p21 by ubiquitination.

### HJURP facilitates HCC proliferation via the MAPK/ERK1/2 and AKT/GSK3β signaling pathways

**Fig. 6:**
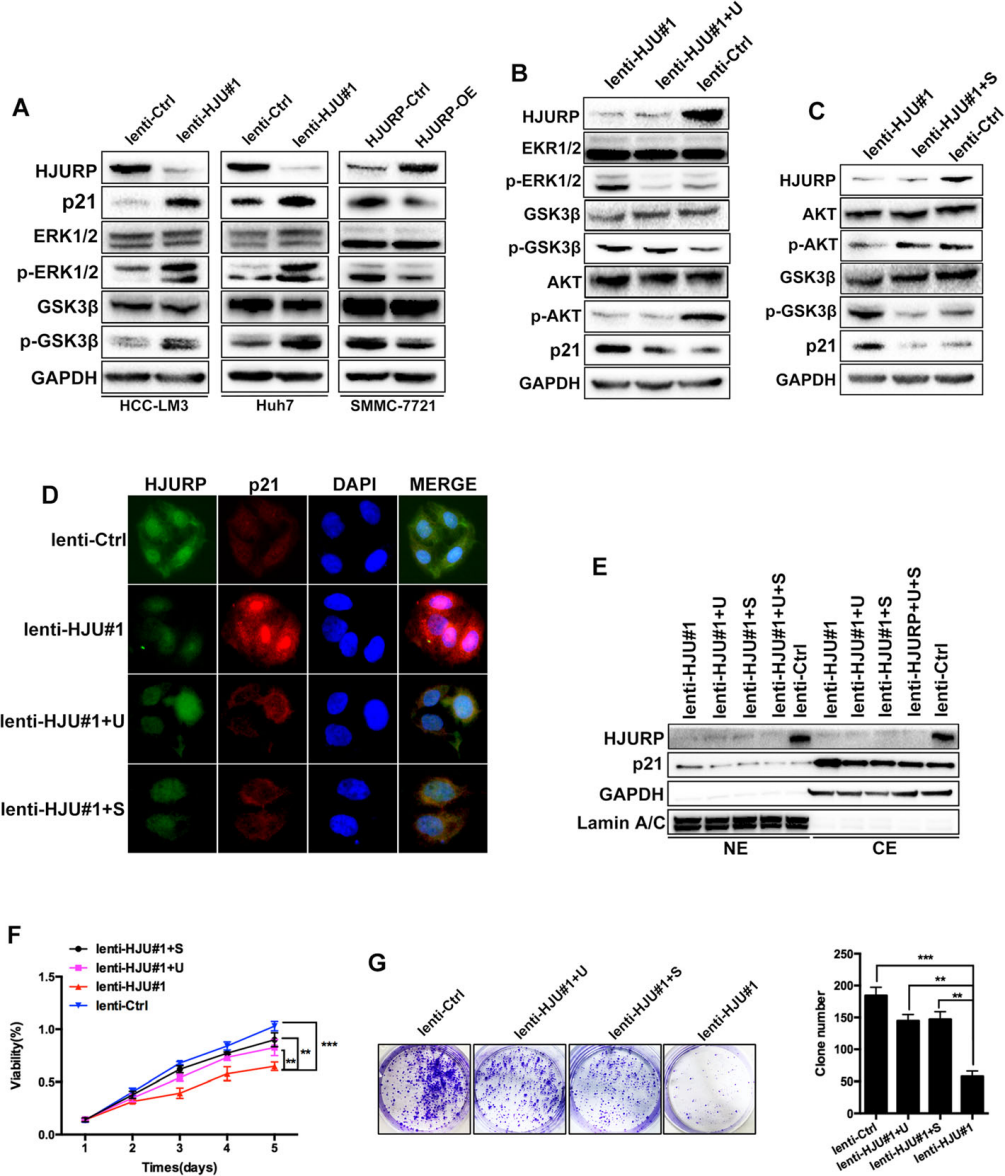
HJURP destabilizes p21 in an ERK1/2 and GSK3β-dependent manner. **a** Protein levels of ERK1/2, p-ERK1/2, GSK3β, p-GSK3β and p21 were detected by western blot in the HJURP knockdown and overexpression cells. **b** HCC-LM3 cells were incubated with the ERK1/2 pathway inhibitor U0126 (5 μM) and **c** AKT activator SC-79 (5 μM) for 24 h before lysis for western blot. ERK1/2, p-ERK1/2, GSK3β, p-GSK3β, AKT, p-AKT were detected by western blot. **d** Immunofluorescence and **e** western blot were used to measure subcellular distribution of p21. U: U0126; S:SC-79. **f** and **g** U0126 and SC-79 reversed the growth inhibition mediated by HJURP-knockdown in HCC-LM3 cells

ERK1/2, GSK3β and JNK are known to be associated with p21 stability [24]. Therefore, we determined whether these three kinases are associated with the regulation of p21 stability by HJURP. Intriguingly, knockdown of HJURP dramatically upregulated the expressions of p-ERK1/2 and p-GSK3β (Fig. 6a, left and middle) but not p-JNK (data not shown). Accordingly, the overexpression of HJURP reduced the expressions of p-ERK1/2, p-GSK3β and p21(Fig. 6a, right). To confirm the involvement of ERK1/2 and GSK3β in the regulation of p21 by HJURP in HCC, we treated HJURP knockdown HCC-LM3 cells with U0126, which is a specific ERK1/2 pathway inhibitor. After 24 h of incubation with U0126 (5 μM), the expression of p21 was suppressed in HJURP knockdown cells. However, U0126 did not reverse the effect on p-GSK3β (Fig. 6b). These results showed that p-ERK1/2 and p-GSK3β may be independently involved in the HJURP-regulated stability of p21. Given that GSK3β acts as a downstream factor of AKT in the PI3K/AKT pathway, we speculated that HJURP might modulate p21 stability by inhibiting the expression of p-GSK3β via the AKT pathway. To test our hypothesis, we added the selective AKT agonist SC-79 (5 μM) to the cell culture medium and incubated the cells for 24 h. Notably, compared with that in cells in the lenti-HJU#1 group, p21 and p-GSK3β, but not p-ERK1/2, expression was reduced in cells in the SC-79 treated group (Fig. 6c).

Using western blot detection, we found that the nuclear and cytoplasmic p21 levels were increased in HJURP knockdown HCC-LM3 cells, whereas these levels were decreased in HJURP-overexpressing SMMC-7721 cells (Fig. 4k). Additionally, U0126 and SC-79 reduced the nuclear accumulation of p21 in the HJURP knockdown HCC-LM3 cells (Fig. 6d, e). Based on the results of the CCK-8 and colony formation assays, growth inhibition was reversed by treatment with U0126 and SC-79 in HJURP knockdown cells (Fig. 6f, g).

Taken together, these findings clearly demonstrate that HJURP modulates HCC cell proliferation by promoting p21 nucleus-cytoplasm translocation via the MAPK/ERK1/2 and AKT/GSK3β pathways.

### High HJURP expression is correlated with low p21 expression and unsatisfactory clinical outcomes in HCC individuals

**Fig. 7:**
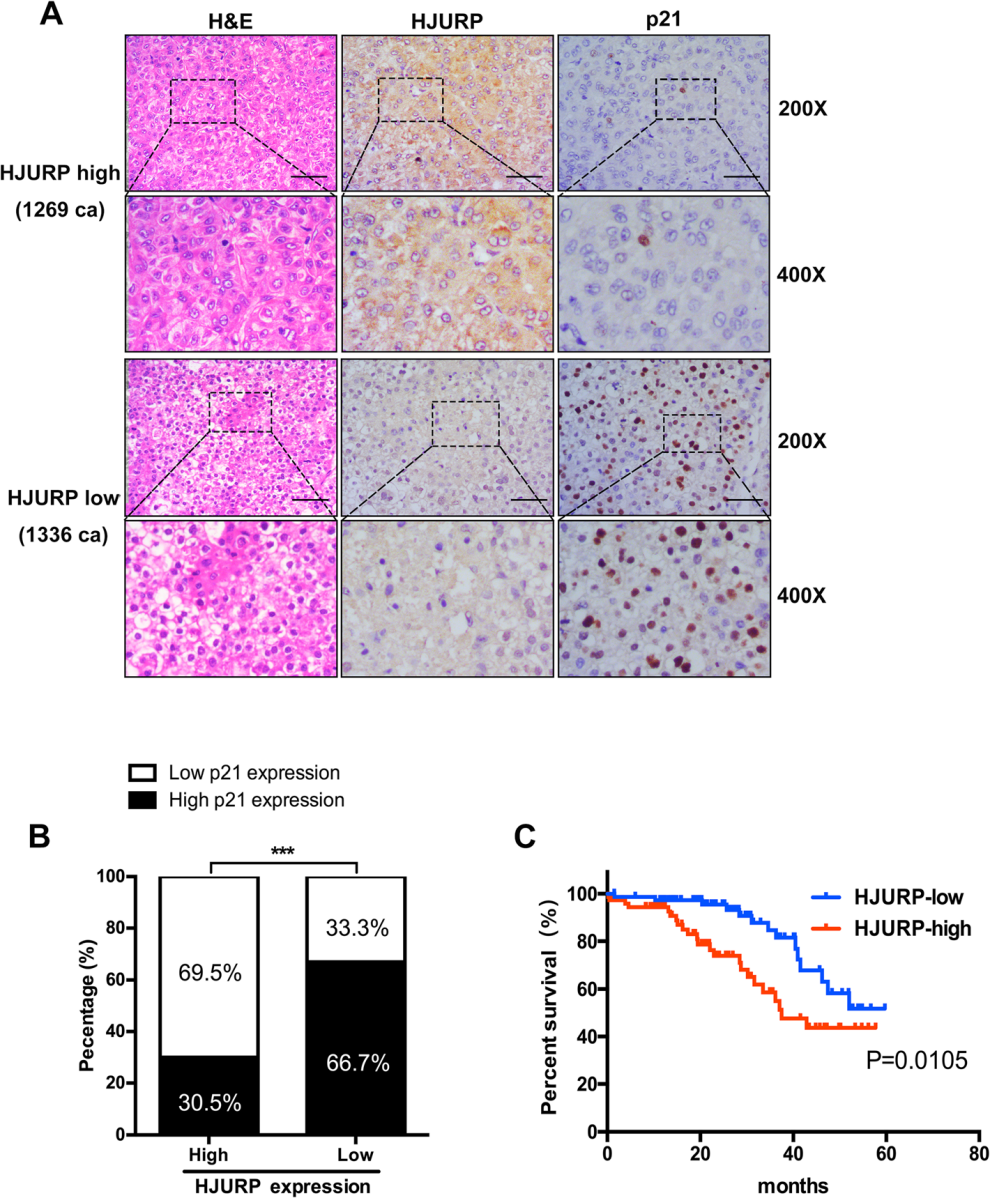
High HJURP expression is correlated with low p21 expression and unfavorable clinical outcomes in HCC individuals. **a** and **b** Immunohistochemical staining of HCC tissues showed that high HJURP expression is correlated with low p21 expression (scale bar: 50 μm; magnification: 200X and 400X). **c** Kaplan-Meier analysis showed that HCC patients with low HJURP expression had a better overall survival rate than patients with high HJURP expression

To examine the clinical significance of p21 dependence, we further performed immunohistochemical assays in HCC specimens to explore the correlation between HJURP and p21(Fig. 7a). Approximately 69.5% of samples with low p21 expression displayed strong staining of HJURP, whereas 30.5% showed weak staining for HJURP. Similarly, 33.3% of samples with high p21 expression displayed weak expression of HJURP, and 66.7% displayed high levels of HJURP (Fig. 7b).

To explore whether HJURP expression was associated with the overall survival rate, we divided 120 HCC patients into two groups based on HJURP density: a high HJURP expression group (*n* = 69) and a low HJURP expression group (*n* = 51). Kaplan-Meier analysis revealed that the patients with low HJURP expression levels had a better overall survival rate than patients with high HJURP expression (Fig. 7c). Furthermore, we analyzed the relationship between HJURP expression and clinicopathologic features of the HCC patients and found significant differences in age, tumor size and tumor stage (Table 1).

**Table 1:** HJURP expression and clinicopathologic features in HCC patients

Variable	HJURP expression		*P* value
	High	Low	
Sex			0.984
Male	65	48	
Female	4	3	
Age (years)			**0.039**
≤ 50	21	25	
>50	48	26	
HBV			0.300
Negative	6	2	
Positive	63	49	
AFP (ng/ml)			0.600
≤ 400	47	37	
>400	22	14	
Cirrhosis			0.130
Yes	59	38	
No	10	13	
Tumor size (cm)			**0.007**
≤ 5	14	22	
>5	55	29	
Tumor number			0.051
= 1	30	27	
>1	39	24	
Tumor stage (AJCC)			**0.003**
I-II	16	25	
III-IV	53	26	
Tumor differentiation			0.306
High	8	2	
Middle	38	29	
Low	23	20	

*AFP* alpha fetoprotein, *AJCC* American Joint Committee on Cancer

*P* values in bold are statistically significant

## Discussion

HJURP has been demonstrated to play an important role in human neoplasms [7–9, 25]. HJURP exhibits oncogenic activity in various cancer types including HCC.

In this study, we found that HJURP promotes HCC cell proliferation both in vivo and in vitro. HJURP was overexpressed in HCC cell lines and clinical specimens. Clinically, HJURP was associated with tumor size and tumor stage. Additionally, Kaplan-Meier analysis revealed that high HJURP expression was correlated with poor overall survival rates in HCC patients. These results suggest that HJURP acts as an oncogene in HCC. In concordance with our findings, Hu et al. reported that HJURP facilitates cell viability in HCC cell lines [9]. However, the underlying mechanism of HJURP-regulated HCC tumorigenicity remains to be elucidated.

We subsequently demonstrated that HJURP knockdown could induced G0/G1 phase arrest but not apoptosis in HCC cells, indicating that HJURP may be a key modulator of cell cycle progression in HCC. p21, a novel tumor suppressor, plays a crucial role in several growth-inhibiting signaling pathways and suppresses cell cycle progression in both p53-dependent and p53-independent manners [26]. We hypothesized that HJURP modulates the cell cycle in HCC by regulating p21. We confirmed that HJURP represses the expression of p21 in HCC cells and clinical samples. Additionally, the phenotypic features driven by HJURP were reversed by p21 knockdown. Given that p21 is mostly modulated at the transcriptional and post-translational levels [13, 27–29], we next explored the mRNA and protein stability of p21. The results of p21 half-life with the addition of significant correlation between HJURP and the proteasome SKP2 implied that HJURP downregulates p21 protein stability at the post-translational level. According to current reports, p21 stability is regulated by a myriad of proteasomal signals, including ERK1/2 and GSK3β. The MAPK/ERK1/2 pathway has been reported to mediate the nuclear localization of p21 in colorectal cancer. Likewise, we demonstrated that HJURP destabilizes p21 via p-ERK and p-AKT respectively. Moreover, we proved that HJURP promotes nucleus-cytoplasm translocation of p21 in HCC cells. Consistent with our findings, Zhang et al. also reported that p-ERK1/2 and p-GSK3β could maintain p21 stability in HCC [24]. However, Zhang and his colleagues indicated that p-ERK1/2 is a key upstream key factor of p-GSK3β. We further validated this hypothesis in HJURP knockdown HCC cells. In contrast to the findings of Zhang and his colleagues, blockage of ERK1/2 with U0126 did not affect the expression of p-GSK3β in our study. Nevertheless, the activation of AKT by SC-79 reversed the stability of p21. In line with our findings, Liu and Gong reported that GSK3β expression is mediated by the PI3K/AKT pathway [30, 31]. Therefore, we propose that in addition to affecting the MAPK/ERK1/2 signaling pathway, HJURP decreases the p21 stability by inhibiting p-GSK3β via the AKT pathway in HCC.

It is well known that the stability of p21 depends on its subcellular localization [14, 32], and nuclear p21 can bind to PCNA to induce anti-proliferative effects. The activation of ERK1/2 is involved in the nuclear localization of p21. Thus, our findings indicate that HJURP destabilizes p21 by reducing its nuclear localization. Four E3 ligases, namely, SKP2, CDT2, LRR1 and CDC20, have been mostly reported to be responsible for p21 degradation. We demonstrated that HJURP is significantly correlated with SKP2 but not with LRR, CDT2 or CDC20. Moreover, we observed that HJURP promotes ubiquitination-mediated p21 degradation. Our results lead to the conjecture that HJURP might promote p21 translocation to the cytoplasm for SKP2-dependent degradation. To the best of our knowledge, this study is the first to reveal that HJURP promotes the ubiquitination and cytoplasmic localization of p21 via the MAPK/ERK1/2 and AKT/GSK3β signaling pathways. In addition to the above four E3 ubiquitin ligases, other proteins can regulate the degradation process of p21. For example, five E3 ubiquitin ligases (UHRF2 [33], UBR5 [34], SOCS1 [35], COPA3 [36] and ZNF313 [26, 37]) and two deubiquitylases (USP11 [38] and USP36 [39]) have been reported to mediate p21 stability. In our work, we investigated four most frequently E3 ligases, especially SKP2. However, other E3 ligases and deubiquitylases may be involved in HJURP-mediated p21 stability. It is worthwhile to continue research to elucidate these unknown factors in our future work.

## Conclusions

**Fig. 8:**
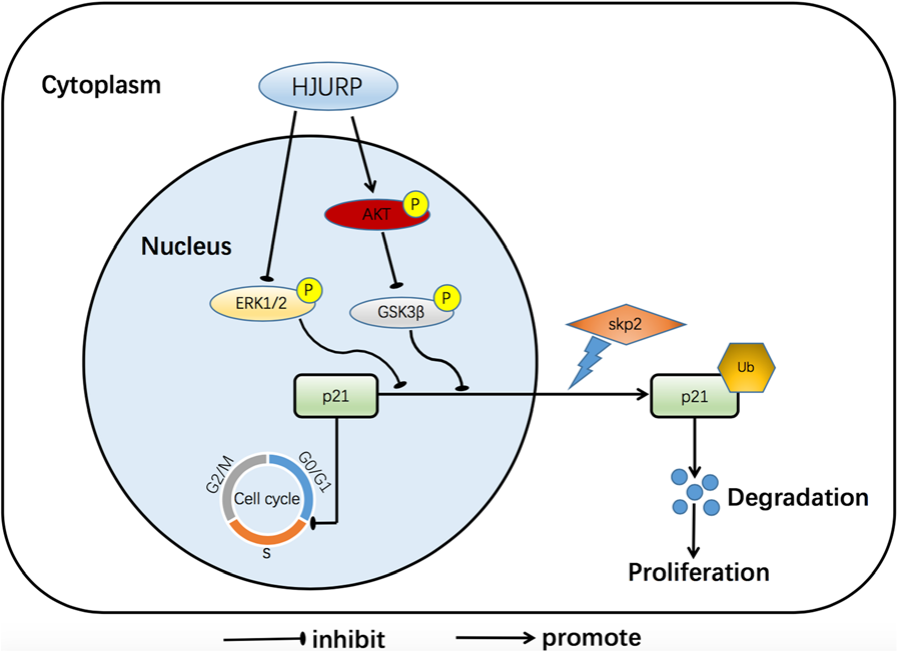
Schematic model representing the function of HJURP in HCC. P: phosphorylation; Ub: ubiquitination

In summary, we have demonstrated that high levels of the expression of HJURP are correlated with poor prognosis in HCC patients and promote HCC cell proliferation via the ubiquitination and cytoplasmic localization of p21 through the MAPK/ERK1/2 and AKT/GSK3β pathways (Fig. 8). This study thus highlights a mechanism by which HJURP promotes HCC growth.

## Abbreviations

ERK1/2: Extracellular signal-regulated kinase 1/2
GSK3β: Glycogen synthase kinase
HCC: Hepatocellular carcinoma
HJURP: Holliday junction recognition protein
JNK: c-Jun N-terminal kinase
